# Wound Healing Activity of *Crassocephalum crepidioides* (Benth.) S. Moore. Leaf Hydroethanolic Extract

**DOI:** 10.1155/2020/2483187

**Published:** 2020-08-22

**Authors:** Nguyen Minh Can, Dang Thi Phuong Thao

**Affiliations:** ^1^Faculty of Biology-Biotechnology, University of Science, Ho Chi Minh City, Vietnam; ^2^Vietnam National University in Ho Chi Minh City, Vietnam; ^3^Department of Biology and Agricultural Engineering, Pham Van Dong University, Quang Ngai, Vietnam

## Abstract

*Crassocephalum crepidioides* (Benth.) S. Moore. has been used to treat small wounds by minority people in Lam Dong, Vietnam. However, there has been no scientific evidences about its wound healing activity. This study is aimed at evaluating the wound healing activity of *Crassocephalum crepidioides* hydroethanolic extract via its antioxidant and anti-inflammation activities and healing capability on a mouse excision wound model. *Crassocephalum crepidioides* hydroethanolic extract (CCLE) at a dose of 50 mg/kg/day reduced the wound closure time about 3.5 days, compared to vehicle treatment. The granulation tissue on day 7 after surgery from the treated group showed a 2.8-fold decrease in the density of inflammatory cells, 1.9-fold increase in the fibroblast density, and a higher number of blood vessels. Real-time PCR analysis indicated that the mRNA expression level of NF-*κ*B1 and TNF-*α* mRNA in CCLE-treated wounds decreased by 4.6 and 3.3 times, respectively, while TGF-*β*1 and VEGF were found to increase by 3.3 and 2.4 times, respectively. Our experimental data provided proofs of *Crassocephalum crepidioides* leaf wound healing activity due to its antioxidant, anti-inflammation, fibroblast proliferation, wound contraction, and angiogenesis effects.

## 1. Introduction

Wound healing is a complex process that often is divided into 3 overlap phases: inflammation, proliferation, and remodeling [1, 2]. The inflammatory phase starts immediately after injury, together with hemostasis. In this phase, leukocytes are recruited to the wound site to remove alien substances and dead tissue and prevent infection [1, 2]. Fibroblasts and keratinocyte proliferation and migration play a key role in the proliferation phase. Fibroblasts are predominant cells at the wound site in this phase, responsible for new matrix and collagen production [2]. The remodeling phase occurs in several months to years, involving change in components of the extracellular matrix at the wound site and restoration of about 80% of tensile strength normal skin [2].

Many traditional medicinal plants (TMPs) have been discovered and used for wound care for centuries. Recent reports suggested that the wound healing mechanism of TMPs might be due to antioxidant, antimicrobial, and anti-inflammatory activities and stimulation on DNA and protein synthesis, epithelium cell proliferation, collagen formation, and angiogenesis [3, 4].

*Crassocephalum crepidioides* (Benth.) S. Moore. (*Compositae* family) is a TMP found in various parts of Asia, Africa, and Australia [5]. The leaves of *C. crepidioides* are traditionally used to treat indigestion, stomachache [5–7], and wound [8, 9]. *C. crepidioides* has been reported to have antibacterial [10], hypoglycemic, antioxidant [11, 12], anti-inflammatory [13, 14], antitumor [14], and antidiabetic [12, 15] activities. Several bioactive compounds have been found in *C. crepidioides* leaves, including some phenolic and flavonoid compounds [11, 16]; the essential oil from *C. crepidioides* leaves contains *β*-cubebene, *α*-farnesene, and *α*-caryophyllene. *C. crepidioides* leaves has been also used for treating wounds in Vietnam, China, India, and some African countries [8, 9, 17]. However, there have been no published materials proving *C. crepidioides* wound healing activity. This study is aimed at investigating the wound healing activity of *C. crepidioides* hydroethanolic leaf extract (CCLE) on a mouse excision wound model, focusing on the wound closure rate, histopathology characteristics, and the mRNA expression level of several genes involved in the wound healing process.

## 2. Materials and Methods

### 2.1. Plant Samples and Preparation of Extract and Fractions

*C. crepidioides* (voucher specimen number PHH0004878) were collected from Bidoup Nui Ba National Park, Lam Dong, Vietnam. The sample was washed by clean water and dried in an oven at 40°C. The dried sample was homogenized to fine powder for further extraction. The dried powder was soaked in ethanol 70% for 48 h (*w*/*v* = 1 : 10) [18, 19]. The solvent is preferred for preparing the extract for bioactivity evaluation purposes because of its low toxicity to cells and animals. The liquid phase then was filtered and dried by a rotary vacuum evaporator until constant weight to obtaine CCLE. The extract was kept at -20°C for later experiments.

### 2.2. Total Phenolic Content

Total phenolic content (TPC) in CCLE was evaluated using a Folin-Ciocalteu colorimetric assay described by Do et al., with slight modification [20]. The sample was dissolved in 70% ethanol solution; 80 *μ*L of the sample was added to 800 *μ*L Folin-Ciocalteu solution (10-fold diluted) in a glass tube, then added 800 *μ*L Na_2_CO_3_ (7.5%) and 320 *μ*L distilled water, mixed thoroughly, and left standing for 30 minutes at room temperature in dark condition. The absorbance of the mixture was measured at 760 nm using a UV-VIS spectrophotometer V-7200 model (Jasco, Tokyo, Japan). TPC was expressed as milligram gallic acid equivalent per gram of CCLE (mg GAE/gE).

### 2.3. Total Flavonoid Content

The total flavonoid content (TFC) CCLE was evaluated according to the method described by Msaada et al. with minor modifications [21]. The CCLE sample was diluted with ethanol 70% solution; 200 *μ*L sample was added to a glass tube containing 120 *μ*L of NaNO_2_ 5%; the mixture was vortexed for 10 seconds and left standing for 5 minutes. Then, 120 *μ*L of AlCl_3_ (10%) and 800 *μ*L of NaOH 1 M were added to the tube. The mixture was then adjusted to 2 mL with distilled water and vortexed for 10 seconds, and absorbance was measured at 415 nm. TFC was expressed as milligram quercetin equivalent per gram of CCLE (mg QE/gE).

### 2.4. DPPH Free Radical-Scavenging Activity

Antioxidant activity of CCLE was investigated by 2,2-diphenyl-1-picrylhydrazyl (DPPH) free radical-scavenging capacity. DPPH 100 *μ*M solution and CCLE were prepared in ethanol 70%; 2 mL of DPPH solution was mixed with 0.5 *μ*L samples at a concentration of 200, 100, 50, 25, and 12.5 *μ*g/mL in a glass tube. The mixture was vortexed and then incubated for 30 minutes in the dark at room temperature. The mixture of 0.5 *μ*L ethanol 70% with 2 mL DPPH 100 *μ*M was used as blank. Absorbance at wavelength 517 nm was measured by spectrophotometer UV-Vis V730. Gallic acid solutions at a concentration of 50, 40, 30, 20, and 10 *μ*g/mL were used as the positive control. The scavenging capacity of CCLE is calculated by the following formula:

(1) $$\begin{array}{c} \text{Scavenging capacity}=\frac{\text{Ao}-\text{Aj}}{\text{Ao}}\times 100\;\left(\%\right), \end{array}$$

where Ao was the absorbance of blank and Aj was the absorbance of the sample.

The IC_50_ value was estimated using GraphPad Prism 7.04.

### 2.5. Cell Culture

Murine macrophage cells RAW 264.7 (ATCC® TIB­71™) were cultured in DMEM-F12 medium supplemented with penicillin and streptomycin (Sigma) and 10% heat-inactivated FBS in 96-well plates, at 37°C and under an atmosphere of 5% CO_2_. The cells were seeded at a density of 10^4^ cells/well with 100 *μ*L medium and subculture every 3 days.

### 2.6. Cell Viability

Toxicity of CCLE on cell RAW 264.7 viability was estimated by a 3-(4,5-dimethylthiazol-2-*y*l)-2,5-diphenyltetrazolium bromide (MTT) assay. Briefly, cells were seeded with 100 *μ*L DMEM-F12 medium supplemented with penicillin and streptomycin (Sigma) and 10% heat-inactivated FBS in 96-well plates at a density of 10^4^ cells/well. After 24 hours, the medium was removed and replaced with fresh medium supplemented with LPS 1 *μ*g/mL; CCLE was dissolved in DMSO 10% in the range of 2 times dilution concentration (from 1000 *μ*g/mL to 15.625 *μ*g/mL) and added to the medium (final concentration of DMSO was 0.1%). After 48 hours of incubation, 10 *μ*L MTT was added to each well, followed by incubation for 3 hours; then, the supernatant was removed and formazan was dissolved with 100 *μ*L isopropanol-HCl; the measured optical density at wavelength is 550 nm. Cell viability was calculated by the following formula:

(2) $$\begin{array}{c} \text{Cell viability }\left(\%\right)=\frac{{\text{OD}}_{\text{sample}}}{{\text{OD}}_{\text{control }\left(\text{without LPS}\right)}}\times 100. \end{array}$$

### 2.7. Anti-inflammation Activity

Anti-inflammation activity was investigated via the amount of nitric oxide (NO) production in the murine macrophage cell RAW 264.7 (ATCC® TIB­71™) induced with lipopolysaccharide (LPS) by the method of Heo et al. [22] with modification. Cells were seeded with 100 *μ*L DMEM-F12 medium supplemented with penicillin and streptomycin (Sigma) and 10% heat-inactivated FBS in 96-well plates at a density of 10^4^ cells/well. After 24 hours, the medium was replaced with fresh medium supplemented with LPS 1 *μ*g/mL with or without CCLE (CCLE was prepared in DMSO 10% and supplemented to medium at a final concentration of 125, 62.5, and 31.25 *μ*g/mL), incubated for 48 hours. Then, 50 *μ*L of cell culture supernatant was mixed with 50 *μ*L of the Griess reagent and incubated at room temperature in darkness for 10 minutes. Absorbance at 550 nm was measured using an ELISA reader.

### 2.8. Wound Healing Activity Evaluation

Male Albino Swiss mice (*Mus musculus*) were purchased from Ho Chi Minh City Pasteur Institute, Vietnam. Mice were kept in laboratory conditions for a week (25°C, with 12 hours of light–dark cycle) and have free access to water and food during the experiment process. The mice (weighing 25 ± 2) were anaesthetized with ketamine (Troy Laboratories) 80 mg/kg and xylazine (Troy Laboratories) 10 mg/kg. To create a wound, the dorsal surface was shaved and then cleaned with ethanol 70%. The excision wound (12 mm: diameter) was made with a biopsy punch (Medax). The mice were divided into 3 groups (*n* = 6). Each mouse was topically treated everyday with 25 *μ*L of CCLE 50 mg/mL and 10 mg/mL, dissolved in DMSO 5%, Tween 20 5% mixture, which is, respectively, equivalent to 50 mg/kg/day (mg/kg/d) and 10 mg/kg/d of CCLE. In the control group, each mouse was treated with 25 *μ*L DMSO 5% and Tween 20 5% mixture in the same way. Photos were taken on the days 4, 8, and 12 after surgery. The wound area was measured by ImageJ 1.50b software. The wound closure rate was calculated by the following formula [23]:

(3) $$\begin{array}{c} \text{Wound closure }\left(\text{day }n\right)\;\left(\%\right)=\frac{\text{wound area at day }0‐\text{wound area at day }n}{\text{wound area at day }0}\times 100. \end{array}$$

### 2.9. Histological Evaluation of the Wounds

After 7 days, the granulation tissues from the 7-day wounds were taken and fixed in formalin solution (10% formalin in phosphate-buffered saline) overnight. The tissue then was dehydrated, cleared, embedded in paraffin by a tissue processing machine, and stained with haematoxylin-eosin (HE). The staining images were taken by a light microscope (BX53, Olympus). Images were analyzed for inflammatory cells, fibroblasts, and blood vessel density using the software ImageJ 1.50b.

### 2.10. RNA Extraction and Quantitative Real-Time PCR

After 7-day treatment, granulation tissues (30 mg) from wounds were collected for RNA extraction, using a TRIzol reagent (Invitrogen). RT-qPCR was used for evaluating mRNA expression of some genes involved in the wound healing process, including tumor necrosis factor *α* (TNF-*α*), nuclear factor kappa-light-chain-enhancer of activated B cells 1 (NF-*κ*B1), transforming growth factor 1 (TGF-*β*1), and vascular endothelial growth factor (VEGF). The glyceraldehyde 3-phosphate dehydrogenase (GAPDH) gene was the housekeeping genes. The primers used for the genes are listed in Table 1.

The reverse transcription reactions were performed with 1 *μ*g total RNA on a total volume of 20 mL using a PrimeScript RT reagent kit (Takara, Japan). The Applied Biosystems 7500 real-time PCR system was used for carrying out and analyzing real-time PCR. Briefly, the reactions were performed in a 20 mL volume using a SYBR Green reaction mix (Takara, Japan) with 2 *μ*L cDNA. The conditions for amplification reaction were 30 s at 95°C, 40 cycles of 30 s at 95°C, and 30 s at 60°C. Gene expression in the treatment group was compared with the control group via the fold change in mRNA expression, which was calculated by the following formula [24]:

(4) $$\begin{array}{c} \text{Fold change}=2^{-\left(\Delta \text{CT\_sample}–\Delta \text{CT\_control}\right)}, \end{array}$$

where

(5) $$\begin{array}{c} \Delta \text{CT\_sample}={\text{CT}}_{\left(\text{target gene of CCLE}‐\text{treated group}\right)}-{\text{CT}}_{\left(\text{GAPDH of CCLE}‐\text{treated group}\right)},\\ \Delta \text{CT\_control}={\text{CT}}_{\left(\text{target gene of control group}\right)}–{\text{CT}}_{\left(\text{GAPDH of control group}\right)}. \end{array}$$

CT is threshold cycle values.

### 2.11. Statistical Analysis

GraphPad Prism 7.04 software was used for analyzing statistical differences (by ANOVA method with Tukey test) and calculating column statistics.

## 3. Results

### 3.1. Total Phenolic Content, Flavonoid Contents, and Antioxidant Activity of CCLE

The hydroethanolic extract from *C. crepidioides* leaves was obtained with a yield about 16.75% of dried weight. Total phenolic content (TPC) was estimated using a Folin-Ciocalteu colorimetric assay. The TPC of CCLE was 114.3 ± 1.7 mg GAE/g CCLE. The total flavonoid content (TFC) of CCLE was 145.46 ± 3.1 mg QE/g CCLE, using the aluminum chloride colorimetric method (Table 2). Antioxidant activity of CCLE was investigated by DPPH free radical-scavenging capacity and showed its IC_50_ as 48.0 *μ*g/mL (Table 2).

### 3.2. CCLE Showed Wound Healing Activity in Mouse Model

**Figure 1:**
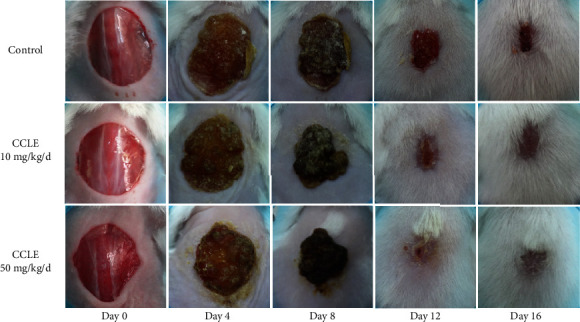
Effect of CCLE on the wound healing process in mice.

**Figure 2:**
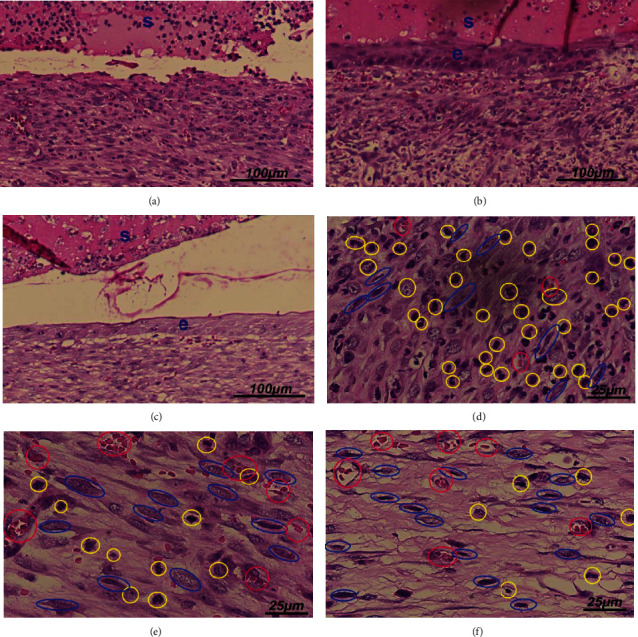
HE staining images of cross-sections of granulation tissues obtained at 7 days postwounding. (a, d) Vehicle-treated group; (b, e) CCLE 10 mg/kg/d-treated group; (c, f) CCLE 50 mg/kg/d-treated group. s: scab; e: epidermis; blue ellipses: fibroblasts; yellow ellipses: inflammatory cells; red ellipses: blood vessels.

Significant differences in the wound closure rate and reepithelialization between CCLE treatment groups and the control group indicated that CCLE have wound healing activity in the mouse model. The results in Table 3 and Figures 1 and 2 showed that CCLE topical treatment dose-dependently increased the wound closure rate. The wound closure percentage in the CCLE 50 mg/kg/d-treated group was significantly higher than that in the control group at an early stage, which was 28.0%, while the percentage in the control group was 15.4% after 4 days. Consequently, CCLE reduced the complete reepithelialization time significantly, which was approximately 3.5 days faster.

In the wound healing process, the inflammatory phase happens immediately after injury and reduces gradually; the proliferation phase starts at late of the inflammatory phase, together with an increase of fibroblasts [25]. In our study, histopathological analysis of the 7-day-old wounds in the CCLE-treated groups showed a decrease in the inflammatory cell density, along with an increase in the fibroblast density and the blood vessel number, compared to that of the control group. Besides, better reepithelialization was also observed in the CCLE-treated wounds (Figure 2). Figures 2(b) and 2(c) showed better epidermis layer formation in CCLE-treated wounds at a dose of 10 mg/kg/d and 50 mg/kg/d, respectively, compared to that of the control (Figure 2(a)). Figures 2(e) and 2(f) indicated that CCLE-treated wounds had less inflammatory cells and more fibroblasts and blood vessels than the nontreatment wounds (Figure 2(d)).

### 3.3. CCLE Anti-inflammatory Activity

**Figure 3:**
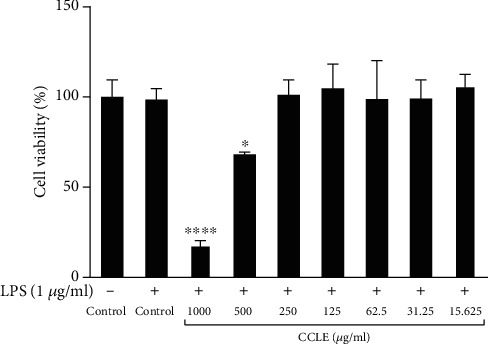
Effect of CCLE on RAW 264.7 cell viability. Vertical columns and bars show data as the mean ± SD.

**Figure 4:**
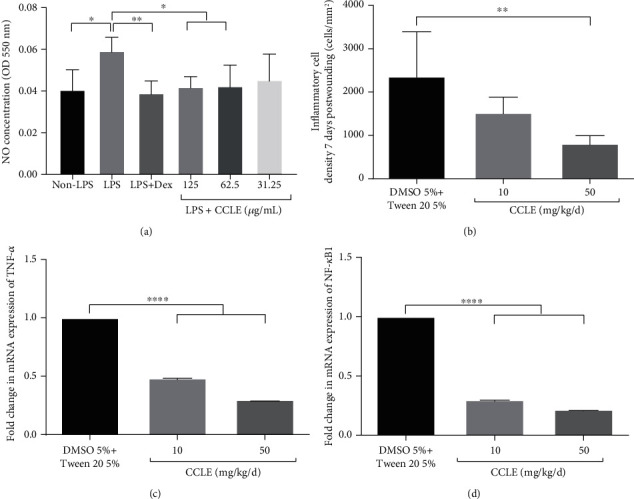
Anti-inflammatory effect of CCLE: (a) NO production inhibition of CCLE in LPS-induced RAW 264.7 cells; LSP was used at a dose of 1 *μ*g/mL; (b) inflammatory cell density; (c, d) the mRNA expression level of TNF-*α* and NF-*κ*B1. Vertical columns and bars showed data as the mean ± SD; ^∗^statistical difference with *p* < 0.05; ^∗∗^*p* < 0.01; ^∗∗∗∗^*p* < 0.0001, compared to vehicle by the ANOVA test.

After the toxicity of CCLE on RAW 264.7 cells was tested, the CCLE concentrations which showed no toxicity to the cells (125, 62.5, and 31.25 *μ*g/mL) were used for anti-inflammatory analysis (Figure 3). CCLE showed anti-inflammatory capacity *in vitro* via the decrease of NO production in the mouse macrophage cell RAW 264.7 induced with LPS 0.1 *μ*g/mL (Figure 4(a)). Anti-inflammation effect of CCLE was dose-dependent, with the highest inhibition of NO production at 91.4% when cells were treated with CCLE at a concentration of 125 *μ*g/mL.

Analysis from the histopathology result of 7-day-old wounds indicated that CCLE treatment at a dose of 50 mg/kg/d reduced the inflammatory cell density by 2.8 times (Figure 4(a)). In addition, CCLE treatment decreased the mRNA expression of TNF-*α* and NF-*κ*B1 dose-dependently. Wounds treated with CCLE 10 mg/kg/d and 50 mg/kg/d had levels of TNF-*α* mRNA lower than the control 2.1 and 3.3 times, respectively (Figure 4(b)). The mRNA expression level of NF-*κ*B1 in the 10 mg/kg/d and 50 mg/kg/d CCLE-treated groups was also downregulated by 3.3 and 4.6 times, respectively, compared to that of the control (Figure 4(c)). Combined with the NO production inhibition in RAW 264.7 cells, the decrease of inflammatory cell density in granulation tissue and the mRNA downregulation of TNF-*α* and NF-*κ*B1 genes suggested that CCLE has anti-inflammatory activity.

### 3.4. CCLE Treatment Increases Fibroblast Density and TGF-*β*1 mRNA Expression

**Figure 5:**
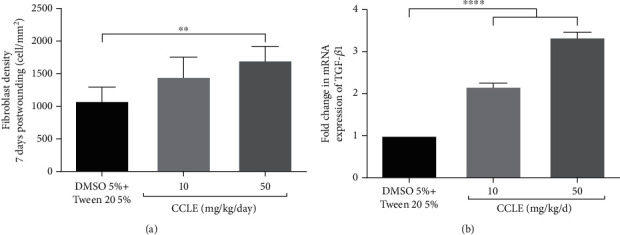
CCLE treatment increased fibroblast density and TGF-*β*1 mRNA expression level in granulation tissue at 7 days postwounding: (a) fibroblast density; (b) TGF-*β*1 mRNA expression level. Vertical columns and bars show data as the mean ± SD; ^∗∗^statistical difference with *p* < 0.01, ^∗∗∗∗^*p* < 0, 0001, compared to vehicle by the ANOVA test.

Fibroblast proliferation is essential to the wound healing process. The HE staining images of 7-day-old wound granulation revealed a significant increase of the fibroblast cell density by 1.9 times in the 50 mg/kg/d CCLE-treated group, compared to that of the control group (Figure 5(a)). Besides, the two CCLE treatment groups also increased TGF-*β*1 gene expression by 2.2 and 3.3 times, respectively, to 10 mg/kg/d and 50 mg/kg/d doses of CCLE (Figure 5(b)). TGF-*β*1 has been reported to be involved in induction on fibroblast proliferation [26–28]. Therefore, the increase of TGF-*β*1 gene expression is strongly correlated with the increase of the fibroblast number in the HE staining images.

### 3.5. CCLE Stimulated Angiogenesis

**Figure 6:**
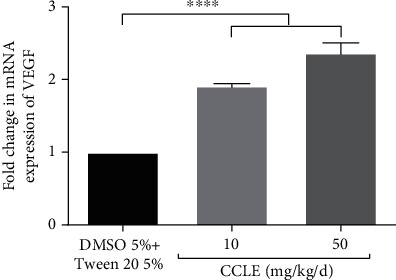
CCLE enhanced the mRNA expression level of VEGF in granulation tissue at 7 days postwounding. Vertical columns and bars show data as the mean ± SD; ^∗∗∗^statistical difference with *p* < 0.001, compared to vehicle by the ANOVA test.

The results in Figure 6 indicated that CCLE has angiogenesis stimulation activity. Wounds from CCLE treatment mice have better neovascularization (Figures 2(e) and 2(f)), compared to the control group (Figure 2(d)). In addition, in the CCLE-treated group at the dose of 50 mg/kg/d, VEGF mRNA expression also significantly increased by 2.4 times, compared to the control (Figure 4).

## 4. Discussion

*C. crepidioides* are traditionally used to treat indigestion, stomachache [5–7], and wound [8, 9]. In this study, we have proven that *C. crepidioides* leaves have wound healing activities.

CCLE showed anti-inflammation in an *in vitro* assay on macrophage cell line RAW 246.7 induced with LPS. It also reduced the density of inflammatory cells in granulation tissues in 7-day-old wounds, combined with a reduced mRNA expression level of TNF-*α* and NF-*κ*B1. NF-*κ*B1 and TNF-*α* are important markers for the inflammation level [24]. TNF-*α* is an important proinflammatory cytokine. NF-*κ*B1 was also reported to be in a direct ratio to the inflammatory level in previous studies [25, 29]. A high level of TNF-*α* has been reported to inhibit the wound reepithelialization and the formation of myofibroblast and *α*-smooth muscle actin (*α*-SMA) [28, 30]. Overinflammation may lead to a delay or failure to heal and may turn into a chronic wound [28]. The decrease in the inflammatory level at the middle and late stages, as well as the decrease of the inflammatory markers, including TNF-*α* and NF-*κ*B1, has been widely considered as a good signal of the healing process [24, 31]. Our results suggested that CCLE might improve the wound healing process via its anti-inflammatory activity. Akinpelu et al. (2019) have proven that water extracts of *C. crepidioides* leaves had anti-inflammation activity via red blood cell membrane stabilization [13]. Bello et al. [32] also found that methanolic extract of *C. crepidioides* had anti-inflammatory activity by lipoxygenase inhibition.

In addition, CCLE treatment increased the fibroblast density at wound sites. TGF-*β*1 mRNA also was found to increase in granulation tissue of the CCLE-treated group. TGF-*β*1 is involved in many important effects on the wound healing process [30, 33]. The activities of TGF-*β*1 include fibroblast proliferation induction [26, 34], motivating fibroblast differentiation into myofibroblast [27], and enhancement of collagen synthesis, deposition, and maturation [35]. Some wound healing agents have been reported to induce TGF-*β*1 expression along with fibroblast proliferation in an animal model [36, 37]. The increase of the TGF-*β*1 gene may explain the increase of fibroblasts and the wound healing effect of CCLE.

Our data indicated that CCLE treatment increased neovascularization in the wounds 7 days after surgery. Moreover, CCLE treatment also increased the VEGF mRNA expression at wound sites. An increase in angiogenesis supports the wound healing process, and delayed or inhibition in angiogenesis impairs wound healing [38, 39]. VEGF is a principal mediator of wound angiogenesis [40]. It has functions not only to promote angiogenesis at several stages during the healing process but also to maintain vessel integrity. VEGF also stimulates the endothelial cell proliferation and increases vascular permeability [39]. The angiogenesis stimulation has been reported in several wound healing remedies and plants; some of them have been reported to induce the expression of VEGF [31, 36, 37]. Since VEGF plays an important role in stimulation of angiogenesis [39], these results may suggest an explanation for angiogenesis stimulation activity of CCLE during the wound healing process.

In addition, a high level of free radical causes oxidative stress to cells, leading to damage of biological material such as protein, DNA, lipid, and tissues [41, 42]. Many wound healing agents showed antioxidant activity [43, 44]. In our study, CCLE showed antioxidant activity via its scavenging capacity to DPPH free radicals. *C. crepidioides* leaf extracts have been also found to showed antioxidant activity in DPPH, 2,2′-azino-bis-(3-ethyl)benzothiazoline-6-sulfonic acid, ferric reducing antioxidant power, and lipid peroxidation assays [12, 13, 32, 45].

Previous reports showed that many wound healing herbs are rich in phenolic and flavonoid contents which play important roles in wound healing activity [4, 25, 36]. These compound effects on the wound healing process might be due to antioxidant, anti-inflammation, antimicrobial, angiogenesis, and cell proliferation stimulation activity [25, 46]. In this study, our results showed that CCLE contains phenolic and flavonoid compounds. Previous studies also indicated that phenolic and flavonoid compounds are present in *C. crepidioides* leaves [13, 45, 47, 48]. Adedayo et al. (2015) found that HCl-methanolic extract of *C. crepidioides* leaves contains many compounds belong to phenolic and flavonoid class, including gallic acid, catechin, chlorogenic acid, caffeic acid, ellagic acid, rutin, and quercetin [45]. Among these compounds, gallic acid, chlorogenic acid, ellagic acid, and rutin have been demonstrated to have wound healing activity [36, 49–52]. These compounds might be relative to wound healing activity of *C. crepidioides* leaves.

Taken together, this study demonstrated a novel aspect of *C. crepidioides* on wound healing. Our data revealed that *C. crepidioides* has wound healing activity. The herb's wound healing effects might support its capacities of antioxidant, anti-inflammation, fibroblast proliferation, and angiogenesis.

## Figures and Tables

**Table 1:** Primers used for RT-PCR.

Gene	Primers (forward/reverse)
GAPDH	5′-AATGTGTCCGTCGTGGATCTGA-3′ 5′-AGTGTAGCCCAAGATGCCCTTC-3′
TNF-*α*	5′-CGAGTGACAAGCCTGTAGCC-3′ 5′-GTGGGTGAGGAGCACGTAGT-3′
NF-*κ*B1	5′-CTGACCTGAGCCTTCTGGAC-3′ 5′-GCAGGCTATTGCTCATCACA-3′
TGF-*β*1	5′-CGCAACAACGCAATCTATG-3′ 5′-ACCAAGGTAACGCCAGGA-3′
VEGF	5′-TCACCAAAGCCAGCACATAGGAGA-3′ 5′-TTACACGTCTGCGGATCTTGGACA-3′

**Table 2:** Phenolic content, flavonoid content, and DPPH scavenging capacity of CCLE.

	TPC (mg GAE/g CCLE)	TFC (mg QE/g CCLE)	DPPH scavenging capacity—IC_50_ (*μ*g/mL)
CCLE	114.3 ± 1.7	145.4 ± 3.1	48.0 ± 1.0
Gallic acid	—	—	2.9 ± 0.0

Data are presented as the mean ± SEM.

**Table 3:** Effect of CCLE on the wound closure rate and reepithelialization time.

Groups	Wound closure percentage (%) at days postwounding			RE time (days)
	4	8	12	
Control	15.4 ± 4.9	46.5 ± 7.7	80.3 ± 7.9	19.7 ± 2.4
CCLE 10 mg/kg/d	21.5 ± 6.0	49 ± 18.7	89.1 ± 20.9	16.8 ± 1.5
CCLE 50 mg/kg/d	28 ± 12.0^∗^	51.4 ± 13.0	92.1 ± 9.5	16.2 ± 1.9^∗^

RE: reepithelialization time. Data are presented as the mean ± SD, *n* = 6. ^∗^Significant difference, compared to the control (DMSO 5%+Tween 20 5%), *p* < 0.05, using unpaired *t*-test.

## Data Availability

The authors state that all data are available.

## Abbreviations

CT: Threshold cycle
CCLE: *Crassocephalum crepidioides* leaf hydroethanolic extract
DMSO: Dimethyl sulfoxide
DPPH: 2,2-Diphenyl-1-picrylhydrazyl
FBS: Fetal bovine serum
GAPDH: Glyceraldehyde 3-phosphate dehydrogenase
LPS: Lipopolysaccharide
MTT: 3-(4,5-Dimethylthiazol-2-*y*l)-2,5-diphenyltetrazolium bromide
NF-*κ*B1: Nuclear factor kappa-light-chain-enhancer of activated B cells 1
OD: Optical density
TGF-*β*1: Transforming growth factor 1
TNF-*α*: Tumor necrosis factor *α*
VEGF: Vascular endothelial growth factor.
